# The Impact of Ozone Treatment in Dynamic Bed Parameters on Changes in Biologically Active Substances of Juniper Berries

**DOI:** 10.1371/journal.pone.0144855

**Published:** 2015-12-14

**Authors:** Agnieszka Joanna Brodowska, Krzysztof Śmigielski, Agnieszka Nowak, Agata Czyżowska, Anna Otlewska

**Affiliations:** 1 Institute of General Food Chemistry, Faculty of Biotechnology and Food Sciences, Lodz University of Technology, Lodz, Poland; 2 Institute of Fermentation Technology and Microbiology, Faculty of Biotechnology and Food Sciences, Lodz University of Technology, Lodz, Poland; National Taiwan University, TAIWAN

## Abstract

The development of the parameters of ozone decontamination method assuring the least possible losses of biologically active substances (essential oils and polyphenols) and their activity in common juniper (*Juniperus communis* (L.)) berries was studied. Ozone treatment in dynamic bed was conducted 9 times. The process was conducted under different ozone concentrations (100.0; 130.0; 160.0 g O_3_/m^3^) and times (30, 60, 90 min). After each decontamination, the microbiological profile of the juniper berries was studied, and the contaminating microflora was identified. Next to the microbiological profile, the phenolic profile, as well as antioxidant activity of extracts and essential oils were determined. The total polyphenol content (TPC), composition of essential oils, free radical-scavenging capacity, total antioxidant capacity, ferric-reducing antioxidant power (FRAP), beta-carotene bleaching test (BCB) and LC-MS polyphenol analysis were carried out. The study reveals that during short ozone contact times, higher amounts of TPC, 15.47 and 12.91 mg CE/g of extract, for samples 100/30 and 130/30, respectively, were demonstrated. Whereas samples 100/60, 130/60, 100/90, and 160/90 exhibited the lowest amount of phenolics. The highest antioxidant activity was found in the methanol extract obtained from ozonated berries which exhibited the lowest IC_50_ in all the antioxidant assays, such as DPPH, FRAP, and BCB assays. Ozone treatment showed noteworthy potential and its usage in food manufacturing and as an alternative decontamination method should be considered.

## Introduction

Spices, due to occurrence in their composition compounds (essential oils, polyphenols) possessing beneficial effects, including antioxidant, as well as anti-inflammatory activity, are an important and integral ingredient of the daily diet [1, 2].

*Juniperus communis* belongs to the family Cupressaceae, and the genus *Juniperus* L., consists of 67 species and 34 varieties varying in size and shape from evergreen tall trees to spreading shrubs [3]. It is widely distributed throughout the Northern Hemisphere. Since antiquity plants from this genus have always been well-known in traditional medicine due to their numerous therapeutic properties such as antiseptic, hypoglycemic, anti-inflammatory, diuretic, hypotensive, anthelmintic, analgesic, and abortifacient [4]. What is more, the chemical composition of the essential oil from juniper berries has attracted attention from medical point of view. Not only essential oil, but also the extract exhibits many biological activities including antidiabetic, anticancer, neuroprotective etc. [5].

*Juniperus* fruits, or female cones, which improperly are called berries, are used as a spice, in northern Europe (Scandinavia), where used to season meat dishes. Nowadays, juniper berries are particularly used for flavoring different alcoholic drinks. For instance, in Dalmatia common juniper (*J*. *communis* L.) is used to prepare a traditional brandy for medicinal purposes [6]. Furthermore, in Serbia there is a type of juniper brandy called “Klekovača”, which possesses its unique aroma. The studies proved that drinking juniper brandy increases appetite. What is more, juniper berries are used in gin production, the Italian liquor “Gineprino”, and the authentic “kozicowe” beer in Poland [4, 7]. In the Polish cuisine juniper berries are well-known to pickle game meat, and are an important ingredient in the traditional Polish dishes such as the cabbage dish “bigos” as well as Polish sausage “kiełbasa jałowcowa” [7].

Because of the mentioned numerous pharmacological properties of extracts and essential oils of juniper berries a proper decontamination method should be chosen, taking into account the biologically active compounds remaining after treatment.

Nowadays, the present health-conscious generation attaches great importance towards the intake of healthy and safe food [2, 8]. The increasing consumption of spices and herbs in industrialized countries requires a proper microbiological purity. Thus, it is necessary to carry out an insightful analysis of spices after each step of the production process. Furthermore, an effective decontamination method of spices should be proposed [9, 10]. In view of the above, we designed the study to evaluate the effectiveness of ozone treatment in a dynamic bed of juniper berries. The reduction of microorganisms, and the content of biologically active substances were taken into account.

## Materials and Methods

### Reagents

2-2-Diphenyl-1-picrylhydrazyl (DPPH); Folin-Ciocalteu’s reagent (2 N); (±)-6-hydroxy-2,5,7,8-tetramethylchromane-2-carboxylic acid (Trolox); (+)-catechin; and 2,4,6-tri(2-pyridyl)-s-triazine (TPTZ), linoleic acid, Tween 40, iron (II) sulfate heptahydrate, iron (III) chloride hexahydrate, β-carotene, RedTaq ReadyMix DNA polymerase, agarose gel electrophoresis and TBE buffer were purchased from Sigma-Aldrich (St. Louis, Missouri, USA). Plate count agar (PCA), DG18 medium, and bullion agar were purchased from Merck (Darmstadt, Germany). The oxidase test kit was obtained from Merck (Darmstadt, Germany). API Tests were purchased from BioMérieux (France). Genomic Mini kit and Clean Up Mini Kit were purchased from A&A Biotechnology (Gdynia, Poland). MJ Mini Gradient Thermal Cycler was obtained from Bio-Rad, (Hercules, CA, USA). BigDye Terminator Ready Reaction Cycle Sequencing kit was purchased from Applied Biosystems (Foster City, CA, USA). Unless indicated otherwise, all chemicals were purchased from Avantor Performance Materials Poland S.A. (Gliwice, Poland).

### Plant Material

Juniper (*Juniperus communis* (L.)) berries were collected in the north-eastern region of Poland–Podlaskie province (52.6500°N, 22.7333°E), and delivered by herbal works KAWON–HURT in Wielkopolska, Poland [11].

### Ozone Treatment in Dynamic Bed

**Fig 1:**
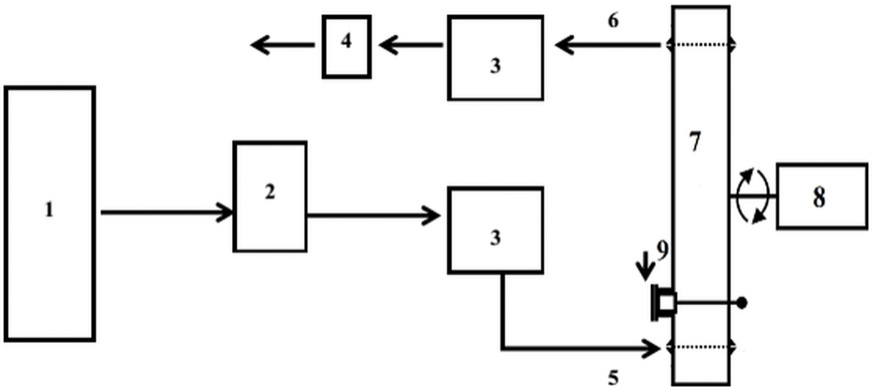
Ozone treatment system in dynamic bed for gaseous phase used for laboratory purposes; 1-oxygen bottle, 2-ozone generator, 3-ozone analyzer, 4- surplus gas elimination unit, 5-inlet of ozone, 6-outlet of ozone, 7-reactor, 8- control system with jolting and rotating mechanism, 9- supply and disposal of plant material treated with ozone.

The procedure of ozone treatment in a dynamic bed (gaseous medium) was performed in a laboratory system consisting of a few basic components such as the gas (pure oxygen), the ozone generator, the electric power source, reactor (cylindrical, glass and steel chambers) directly connected with control system with jolting and rotating mechanism, the surplus gas elimination unit, and the ozone analyzer (Fig 1) [12]. Ozone, previously generated from an oxygen bottle by a laboratory Ozone Generator BMT 803 N (BMT Messtechnik Berlin, Germany), was transferred to the reactor in which was placed the contaminated sample (60.0 g each). The control system with jolting and rotating mechanism allowed to operate the ozone treatment process. The ozone analyzer BMT 964 (BMT Messtechnik Berlin, Germany) was used to determine ozone concentrations both at the inlet and outlet. Samples were labelled as ozone concentration/time, for instance 100/30, 130/30 etc. and were treated with ozone under conditions as follows: ozone concentrations 100.0; 130.0; 160.0 g O_3_/m^3^; times of the process 30; 60; 90 min. The flow rate and pressure was kept constant at 0.1 L/min and 0.5 atm, respectively. After decontamination, each sample was transferred to sterile packaging. Moreover, an ozone sensor (Eco Sensors Model A-21ZX, Newark, USA) was installed to be able to keep track of ozone concentration in the laboratory.

### Microbiological Analysis

The samples of common juniper were prepared according to ISO 6887–4. Total mesophilic bacteria count (TMC) and total bacterial spore count (TSC) were determined on plate count agar (PCA) medium (incubation at 30°C, aerobically). For the determination of the amount of bacterial spores, a thermal shock (80°C, 10 min.) was provided at the initial suspension. The *Enterobacteriaceae* count (EE) was determined on VRBG agar followed by incubation at 30°C for 24 h. The total count of fungi (TFC) included isolation, culturing on Czapek-Dox agar and incubation at 25°C for 7 days. The colonies were counted as colony-forming units (cfu) per gram of sample. The results were presented as mean ± standard deviation.

The dominant bacteria were selected and identified by standard methods based on morphological features, Gram staining, oxidase and catalase tests, glucose metabolism, endospore formation, motility, using standard procedures [13] and biochemical assays using 50 CHB API tests (bioMerieux, France). Oxidation or fermentation of glucose were examined in Hugh Leifson (HL) medium after incubation for 72 h at 30°C. The species belonging to the same taxonomical class were further identified. The taxonomic classification of bacteria was confirmed by molecular methods based on 16S rRNA gene sequencing. Genomic DNAs were extracted using the Genomic Mini kit (A&A Biotechnology, Gdynia, Poland), according to manufacturer’s instruction. The amplification of bacterial 16S rRNA genes were performed with universal primers 27f (5’-AGAGTTTGATCCTGGCTCAG-3’) and 1492r (5’-GGTTACCTTGTTACGACTT-3’) in the MJ Mini Gradient Thermal Cycler (Bio-Rad, Hercules, CA, USA). Each PCR reaction was carried out in 50 μl volume containing 40 pmol of each primer, 1.5 U of RedTaq ReadyMix DNA polymerase (Sigma-Aldrich, St. Louis, MO, USA), 20 ng of template DNA and made up to 50μl with PCR grade water. The PCR was run using the following thermal cycling program: initial denaturation at 94°C for 2 min, 34 cycles consisting of denaturation at 94°C for 1 min, primer annealing at 50°C for 1 min and elongation at 72°C for 2 min, and a final extension step at 72°C for 2 min. PCR products were detected by 1% (w/v) agarose gel electrophoresis in 0.5 × TBE buffer (Sigma-Aldrich, St. Louis, MO, USA) and purified using Clean Up Mini Kit (A&A Biotechnology, Gdynia, Poland) following the manufacturer’s purification protocol. The nucleotide sequences of the 16S rRNA genes were obtained using the BigDye Terminator Ready Reaction Cycle Sequencing kit (Applied Biosystems, Foster City, CA, USA). The reaction products were analyzed using an Applied Biosystem model 3730 Genetic Analyzer (Genomed, Warsaw, Poland). The nucleotide sequences of 16S rRNA were proofread, assembled and compared with sequences available in The National Center for Biotechnology Information (NCBI) using blastn algorithm (BLASTN 2.2.30+) [14]. The nucleotide sequences of 16S rRNA genes were deposited in the NCBI GenBank database with the accession numbers: KP676166 (*Bacillus subtilis* 2J), KP676167 (*Bacillus pumilus* 3J), KP676168 (*Bacillus cereus* 4J). Fungal colonies (1J) were microscopically visualized and identified by morphological traits.

### Isolation of Essential Oil

Ground berries of juniper (*J*. *communis* (L.)) (40.0 g) before (control) as well as after ozone treatment were immersed in 600.0 mL water in a round-bottom flask. The essential oil was obtained by 3-hours-continuous hydrodistillation of juniper in an apparatus—the modification of Deryng instrument for analytical isolation of essential oil by hydrodistillation—constructed in the Institute of General Food Chemistry [15]. This apparatus in particular does not release any odors and separates phases very well.

### Gas Chromatography–Mass Spectrometry (GC-MS) of Essential Oils

Essential oils were analyzed according to a previously published procedure [16]. GC–MS analyses were carried out using a Trace GC Ultra gas chromatograph connected with a DSQ II mass spectrometer (Thermo Electron Corp., Waltham, Ma., U.S.A.). Chromatographic separations were performed on Rtx-1 nonpolar capillary column (30 m × 0.32 mm; 0.25 μm film thickness). The temperature program for Rtx-1: 60 to 300°C at 4°C/min. The injector (SSL) temperature was 280°C, and transfer line temperature 200°C. He was used as the carrier gas, flow rate 1 mL/min, split ratio 1:20. The identification of the essential oil components was based on a comparison of their retention indexes (RI), mass spectra (NIST and Wiley libraries), and literature data [16, 17, 18].

### Preparation of Extracts

Juniper berry extracts were obtained by triple extraction of 0.5 g of the material (treated as well as not treated with ozone), which were ground before, with 4.0 mL 70% methanol. Then, sample was mixed by vortex (1 min) and placed into an ultrasonic bath (InterSonic S.C., Olsztyn, Poland) (10 min). The mixture was centrifuged (Labofuge 300, ThermoScientific, Waltham, MA, USA) at 2500 rpm for 5 minutes at room temperature. The supernatant was decanted (10 mL volumetric flask) and the next portion of extractant (3.0 mL 70% methanol) was added to residue. The above steps were repeated twice. All three supernatants were mixed and diluted till the mark (10 ml). The obtained extracts (10 mL each) were recovered and stored in the refrigerator until analysis.

### Determination of Total Polyphenol Content (TPC)

Estimation of the total polyphenol content (TPC) in the extracts was done following the Folin-Ciocalteu procedure [19] taking the modification of Singleton and Rossi (1965) [20] into account. The absorbance was recorded at 720 nm using a Hewlett Packard 8453 Spectrophotometer (Waldbronn, Germany). A standard curve was prepared using different concentrations (30.0–180 μg/mL) of catechin. The results were expressed as mg catechin equivalent per g extract (CE/ g).

### LC-MS^n^ Analysis of Phenolics

Samples of *J*. *communis* (L.) extracts were 2-fold concentrated by a rotary evaporator (IKA RV10C S99; Staufen, Germany), dissolved in 5 mL of 70% methanol and filtered through a 0.22 μm membrane prior to analysis. Determination of phenolics was carried out according to a previously published procedure [16]. Samples were injected onto the high-performance liquid chromatograph (HPLC) column. The HPLC was coupled on-line with an MS LTQ Velos mass spectrometer (ThermoScientific). Chromatographic separation was achieved with a column operated at 45°C. The mobile phase consisted of solvent A (1 mL formic acid in 1 L deionized water) and solvent B (95% acetonitrile). The column used was a Hypersil Gold 150 × 2.1, particle size 1.9 *μ*m (ThermoScientific). The elution began with 96% to 85% A for 8 min, continued with 85% to 82% A for 12 min, from 82% to 60% A for 40 min, from 60% to 50% A for 4 min, the same for 3 min, from 50% to 96% A for 2 min, followed by washing and re-equilibration of the column. Mass spectra were recorded within 85 min. The injection volume was 10 *μ*L. The flow rate was set at 220 *μ*L/min. Electrospray ionization mass spectrometry was performed using a LTQ Velos mass spectrometer (ThermoScientific) equipped with an ESI interface and controlled by Excalibur software. Mass spectra were acquired in negative mode over the range *m/z* 120 to 1000. The I spray voltage was 4 kV. The sheath gas flow rate was 25 and auxiliary gas flow rate was 10. The desolvation temperature was 280°C, and the source temperature was 350°C. Peak identification was performed by comparison of the retention time and mass spectra of standards [16].

### Antioxidant Activity

#### Total antioxidant capacity by DPPH assay

The total antioxidant capacity of the methanolic extracts as well as the essential oils was determined spectrophotometrically, following the modified procedure described by Hatano et al. (1988) [21] taking into account the method of Brand-Williams et al. (1995) [22]. The absorbance was measured after 30 min, using a spectrophotometer (Hewlett Packard 8453; Waldbronn, Germany) at 515 nm and quantified using Trolox (15.0–240.0 mg/L) as a standard. The results were expressed as mg Trolox equivalent TE/ g of a sample. Inhibition of DPPH radical was measured as the decrease in absorbance of the samples versus DPPH standard solution. Lower absorbance of the reaction mixture indicated higher free radical-scavenging activity. The antiradical activity was expressed as IC_50_ (μg/L); this is the extract concentration necessary to scavenge 50% DPPH free radicals. The determination was carried out in triplicate, and the results were expressed as mean values ± standard deviation (SD).

#### Ferric-reducing antioxidant power assay (FRAP)

The ferric-reducing antioxidant power (FRAP) of the essential oils and extracts of juniper berries was tested following the assay of Oyaizu (1986) [23], and taking into account the Benzie and Strain (1996) [24] procedure. The FRAP assay measures the change in absorbance at 593 nm owing to the formation of a blue-colored Fe^II^-tripyridyltriazine compound from the colorless oxidized Fe^III^ form by the action of electron-donating antioxidants. A standard curve was prepared using different concentrations (0.1–1.0 mM) of FeSO_4_ × 7 H_2_O. The results were expressed as mmol FeSO_4_ × 7 H_2_O equivalent.

#### β-carotene bleaching test (BCB)

The β-carotene bleaching test (BCB) of the methanolic extracts as well as the essential oils of juniper berries was determined according to slightly modified procedure of the β-carotene bleaching [25]. The absorbance was measured after two-hours-incubation at 50°C at 470 nm using a Hewlett Packard 8453 spectrophotometer (Waldbronn, Germany). The percentage inhibition of β-carotene was calculated from the data with the slightly modified formula [26]:

$$\%\;inhibition=\left[\frac{A_{A(120)}-A_{C(120)}}{A_{C(0)}-A_{C(120)}}\right]*100\%$$

where A_A(120)_ is the absorbance of the antioxidant at t = 120 min, A_C(120)_ is the absorbance of the control at t = 120 min, and A_C(0)_ is the absorbance of the control at t = 0 min.

### Statistical Analysis

All determinations were carried out in triplicate. Mean values with standard deviations (±SD) were reported for each case. Statistical analysis (means, standard deviation) and analysis of variance (One-Way ANOVA) were conducted using OriginPro 8., Microcal, Northampton, MA, USA, 2007.

## Results and Discussion

### Microbiological Analysis and Changes during Ozone Treatment

Actually, the European Union does not specify the maximum contamination level of spices. Several countries have laid out some specifications for microbial parameters in spices, for instance Germany set up maximum limits of 10^5^, 10^4^, and 10^2^ cfu of total aerobic mesophilic bacteria, *Bacillus cereus* and *Staphylococcus aureus*, respectively, per gram spice. Additionally, *Escherichia coli* should not be present, and the *Salmonella* count should be zero in 25-g sample of spice [13, 27]. What is more, the International Commission on Microbiological Specifications for Foods established standard values for total aerobic mesophilic bacteria, yeasts and molds, coliforms and *E*. *coli*, which are 10^6^, 10^4^, 10^4^, and 10^3^, cfu, respectively, per gram spice [28]. Summarizing, the microbiological contamination of spices should be low enough to ensure safety for consumers.

The results of the microbial analysis of juniper berry samples are summarized in Table 1. The control samples indicated a high level of contamination: the total mesophilic bacteria as well as the total fungal counts were unacceptably high (>10^5^ cfu/g). Our results confirmed those obtained by Garrido et al. (1992) [29], where mold contamination in stick and ground cinnamon, capers, saffron, badian, cardamom, juniper, fennel, artemisia, bay leaf, mint, parsley, rock tea, and ground mustard seed was extremely high (10^5^ cfu/g). However, in relation to the results of the *Enterobacteriaceae* count, the relatively low occurrence of EE indicates the hygienic quality of juniper berries [13].

**Table 1:** Occurrence of microbial contamination in common juniper (*J*. *communis* (L.)) berries after ozone treatments.

Ozone treatment	Total mesophilic bacteria count	Total fungal count	*Enterobacteriaceae* count
control	5.2 ± 0.05^d^	5.2 ± 0.06^c^	< 1*
100/30	4.6 ± 0.12^c^	4.7 ± 0.07^b^	< 1*
130/30	3.8 ± 0.01^a^	4.2 ± 0.11^a^	< 1*
160/30	4.2 ± 0.03^b^	4.4 ± 0.07^a^	< 1*
control	5.1 ± 0.10^a^	5.5 ± 0.04^b^	< 1*
100/60	5.5 ± 0.04^b^	6.0 ± 0.19^d^	< 1*
130/60	5.4 ± 0.03^b^	5.3 ± 0.10^c^	< 1*
160/60	5.0 ± 0.03^a^	4.9 ± 0.14^a^	< 1*
control	6.1 ± 0.10^c^	5.8 ± 0.02^c^	< 1*
100/90	5.3 ± 0.03^b^	5.2 ± 0.02^b^	< 1*
130/90	4.5 ± 0.08^a^	4.2 ± 0.11^a^	< 1*
160/90	4.6 ± 0.02^a^	4.4 ± 0.12^a^	< 1*

All results are given as log (cfu g^-1^). The results obtained were expressed as mean ± SD with n = 3 according to One-Way ANOVA. Different letters (a-d) in columns designate statistically significant differences between different ozone doses at the same time (P < 0.05).

*not detected at the level 10 cfu per 1 gram

The investigated study indicated that samples of common juniper berries treated with the different doses of ozone retained their color compared to the control sample (not treated with ozone). Similarly, Akbas and Ozdemir (2006) [30] observed no significant changes in color of pistachios after being treated with ozone at 0.1 and 1.0 ppm for up to 360 min. The same observations were confirmed in the study of the decontamination of dried figs and cardamom seeds in which no color change was observed after ozone treatment [16, 31].

Ozone treatments (100.0; 130.0; 160.0 g O_3_/m^3^) were carried out at three different contact times (30; 60; 90 min) to develop the most appropriate parameters of decontamination method against microorganisms in common juniper. The obtained results show that the contamination of seeds with mesophilic bacteria and total fungal count varied during ozone treatment from 10^5^ cfu/g to 10^3^ cfu/g. Also, members of *Enterobacteriaceae* were not detected, before and after ozone treatment. There were statistically significant differences at P < 0.05 level between the total mesophilic count in control samples and those treated with ozone for 30 min. It was shown that 30 min of ozone treatment was sufficient to reduce the count of mesophilic bacteria. The sample of juniper berries treated with 130 g O_3_/m^3^ during 30 min indicated a 1.4 log reduction in TMC. The ozone was effective in reducing TFC during short contact with spices (130/30; 160/30), a 1.0 log reduction was observed. However, the study shows that a longer time of ozone treatment (60 min) causes activation of bacteria, higher level of TMC in the range 5.4–5.5 log cfu/g was observed in samples after ozone treatment than in control ones (5.1± 0.10 log cfu/g). It can be explained by a heterogeneous sensitivity of microorganisms to ozone, which may be related to differences in the construction of bacterial walls, or may be further influenced by many factors [32, 33, 34, 35, 36]. The significant differences were observed in the samples treated with ozone in 90 min–a 0.8; 1.6; and 1.5 log reduction in TMC was observed for 100; 130, and 160 g O_3_/m^3^, respectively. The same conditions of ozone treatment resulted in a 0.6; 1.6; and 1.4 log reduction in TFC, respectively. To conclude, a short contact time (30 min) and a high dose of ozone (130 g O_3_/m^3^) are effective enough to reduce TMC and TFC. However, during a 60-minute ozone treatment, an activation of bacteria and fungi is observed, which means that those species may become resistant to ozone exposure. Therefore, in this study a longer contact with ozone (90 min) was used to check an effectiveness. But, the obtained results of a longer ozone treatment time (90 min) show similar effectiveness as after 30 min. It may be explained by various sensitivity microorganisms to ozone. Fungi are more resistant than bacteria (Gram-positive and Gram-negative bacteria) [33]. Besides, the study was conducted on a heterogeneous matrix (juniper berries), and hence the used procedure requires a lot of advanced research to improve it.

The dominant bacteria were isolated and identified using morphological features, Gram staining, oxidase and catalase tests, glucose metabolism, endospore formation, motility, using standard procedures. Furthermore, biochemical assays were conducted using 50 CHB API tests. The species belonging to the same taxonomical class have been further identified using molecular methods. Dominant bacteria in the samples before as well as after ozone treatments included *Bacillus subtilis*, *Bacillus pumilus*, and *Bacillus cereus*. The detected bacteria species are presented in Table 2. Predominant bacteria included *Bacillus subtilis*, were found in 70.0% and in the range 60.0–75.0% of the analyzed samples before and after ozone treatments, respectively. The following occurrence of other bacterial species was noticed: *Bacillus pumilus* (in 10.0% of control samples and in the range 10.0–25.0% of samples after ozone treatments), and *Bacillus cereus* (in 20.0% of control samples and in the range 5.0–25.0% of samples after ozone treatments).

**Table 2:** Detection of the most dominant bacterial species of juniper (*J*. *communis* (L.)) berries after ozone treatments.

Ozone treatment	Percentage distribution of species		
	*Bacillus subtilis*	*Bacillus pumilus*	*Bacillus cereus*
control	70	10	20
100/30	70	15	15
130/30	60	20	20
160/30	60	15	25
100/60	65	20	15
130/60	65	25	5
160/60	75	15	15
100/90	75	20	5
130/90	65	20	10
160/90	65	25	5

According to EFSA the infective dose of *B*. *cereus* is 10^5^–10^6^ cfu. Taking into account our results, the contamination of juniper berries in the control sample was at the level of 1.7 x 10^5^ cfu/g. After the ozone treatments the contamination level of *B*. *cereus* was at the level of 10^5^ to 10^3^ cfu, but had a small percentage distribution (predominatingly 5.0–15.0% of the tested samples). The food is considered as safe if the level of contamination is 10 times lower than the infective dose. Thus, although *Bacillus cereus* is harmful to humans and causes foodborne illness, after contact with the highest ozone doses it occurred at the safe contamination level [37].

There was one dominant fungal species found in 100% of analyzed samples before as well as after ozone treatments. The identification by morphological traits (microscopic and macroscopic visualizations) allows to classify the detected species to the genus *Eupenicillium cinnamopurpureum*. It originates from soil, and often becomes associated during seed colonization. Additionally, *E*. *cinnamopurpureum* occurs in flour, cereals, dried food, as well as fodder. Its growth may be possible at a lower water activity [38].

The spice contamination level may be associated with microorganisms naturally occurring in their environment as well as microorganisms from soil, air, or water during harvesting, drying, transporting, and storage [16, 39].

### Essential Oil Characterization

The composition of the essential oils (EOs) of berries (treated and non-treated with ozone) of *J*. *communis* (L.) from Wielkopolska (Poland) are shown in Table 3. The essential oil profile from juniper berries contains more than 60 compounds, out of which 45 were identified, contributing to 82.91–93.76% of the total essential oil content of berries treated and non-treated with ozone. The essential oil yield ranges from 0.99% to 1.72%, and does not indicate statistical differences at P < 0.05 level. The results were compared with the retention indices (RIs) of authentic samples and their mass spectra with those of standard libraries (NIST and Wiley) and the literature [17, 18]. The monoterpene hydrocarbons presented the main compounds of the EOs, with α-pinene (17.48–41.93%) as the most dominant one. Furthermore, in somewhat lower, but noticeable amounts, 9.58–13.74% for essential oil of juniper berries before and after ozone treatments, respectively, with β-myrcene. Other monoterpens presented in a moderate percentage were sabinene (2.73–4.64%), β-pinene (1.81–2.53%), and limonene (3.75–5.14%). The main oxygenated monoterpene was terpinen-4-ol which varied from 4.23% in control sample to 2.51–3.94% in samples after ozone treatments. The most dominant sesquiterpene hydrocarbons were presented in amounts 2.92–5.53%, 3.64–7.39%, and 1.97–3.94%, with (E)-caryophyllene, germacrene D, and α–humulene, respectively. In most references, α-pinene was also found to be the most dominant, as well as in smaller amounts other terpens such as β-myrcene, germacrene D, sabinene occurred [7, 40]. Comparison between the chemical composition of *J*. *communis* (L.) oils in the control sample and the treated ones showed no differences in their quality. However, there were significant differences in quantity of some constituents. The greater losses can be observed for the highest ozone doses (160, 130 g O_3_/m^3^) and the longest times of treatment (90 min), where there is almost 50% lower amount of α-pinene (19.88%; 17.48%) compared to the control sample (32.65%). To our knowledge, the chemical composition of the essential oils from ozonated and not ozonated juniper berries is reported here for the first time. Nevertheless, these differences came as no surprise, as it is well-known that ozone is a strong oxidant [16]. Therefore, such dispersion may be explained by alkylation of for instance–CH_3_ groups in α-pinene during long contact with ozone. On the other hand, if we take into account groups such as methylidyne ≡CH, or methylene = CH_2_ occurring for instance in (E)-caryophyllene, it can be supposed that its triple, or double bond can be broken, and then oxygen molecule is attached resulting in peroxides. Thus, there were demonstrated higher percentage in such kind of compounds.

**Table 3:** Comparison of the composition of essential oil of juniper (*J*. *communis* (L.)) berries obtained after ozone treatments.

			Control	After ozone treatment								
				100/30	130/30	160/30	100/60	130/60	160/60	100/90	130/90	160/90
**No**	**Compounds**	**RI** ^a^	**%**									
**1**	α-Thujene	926	0.64±0.09	0.78±0.11	0.80±0.08	0.73±0.09	0.63±0.12	0.68±0.05	0.67±0.11	0.72±0.06	0.41*±0.02	0.37*±0.08
**2**	α-Pinene	936	32.65±2.23	41.93*±0.79	38.70*±2.85	35.65±1.56	32.42±0.16	34.26±0.45	33.51±0.22	32.70±0.46	19.88*±0.37	17.48*±0.90
**3**	Camphene	946	0.31±0.08	0.35±0.02	0.35±0.00	0.33±0.02	0.31±0.00	0.27±0.01	0.32±0.01	0.31±0.02	0.23±0.01	0.20*±0.02
**4**	Sabinene	969	3.86±0.33	4.64*±0.10	4.38*±0.08	4.15±0.05	4.04±0.02	4.17±0.06	4.04±0.03	4.28*±0.12	2.73*±0.07	2.85*±0.11
**5**	β-Pinene	973	2.53±0.08	2.49±0.23	2.46±0.18	2.41±0.10	2.32±0.13	2.28*±0.11	2.28*±0.09	2.40±0.14	1.81*±0.03	1.81*±0.06
**6**	β-Myrcene	987	13.74±1.26	12.98±0.19	13.28±0.23	12.19*±0.15	12.97±0.57	13.56±0.08	13.14±0.04	12.93±0.63	8.90*±0.17	9.58*±0.10
**7**	α-Terpinene	1009	0.46±0.04	0.30*±0.02	0.42±0.02	0.43±0.02	0.38±0.04	0.34*±0.03	0.39±0.03	0.34*±0.03	0.29*±0.01	0.34*±0.00
**8**	β-Cymene	1013	0.66±0.05	0.49*±0.03	0.56±0.05	0.60±0.03	0.56±0.05	0.55±0.06	0.58±0.04	0.52*±0.03	0.59±0.04	0.62±0.04
**9**	Limonene	1024	5.14±0.26	4.24*±0.19	4.62±0.39	4.51±0.40	5.09±0.13	4.71±0.27	4.72±0.16	4.23*±0.11	3.75*±0.09	4.56±0.28
**10**	γ-Terpinene	1052	0.83±0.07	0.55*±0.03	0.75±0.02	0.76±0.01	0.70±0.06	0.65*±0.05	0.70±0.07	0.60*±0.05	0.59*±0.03	0.70±0.06
**11**	α-Terpinolene	1081	0.74±0.06	0.56*±0.02	0.66±0.04	0.68±0.01	0.69±0.02	0.64±0.04	0.68±0.04	0.61±0.05	0.61±0.05	0.66±0.04
**12**	Linalool	1085	0.18±0.05	0.20±0.02	0.15±0.01	0.19±0.00	0.20±0.01	0.16±0.01	0.21±0.02	0.21±0.00	0.19±0.01	0.28±0.05
**13**	α-Campholenal	1103	0.34±0.06	0.25±0.05	0.24*±0.02	0.28±0.02	0.27±0.02	0.28±0.01	0.30±0.00	0.27±0.02	0.39±0.03	0.42±0.03
**14**	*cis*-p-Menth-2-en-1-ol	1107	0.13±0.01	0.09*±0.00	0.09*±0.01	0.12±0.01	0.11±0.02	0.11±0.01	0.10±0.02	0.11±0.01	0.14±0.02	0.15±0.01
**15**	*trans*-Pinocarveol	1124	0.45±0.04	0.26*±0.01	0.25*±0.02	0.30*±0.00	0.29*±0.05	0.31*±0.01	0.31*±0.01	0.38±0.03	0.51±0.04	0.56*±0.05
**16**	*cis*-Verbenol	1129	0.43±0.03	0.15*±0.00	0.12*±0.01	0.33±0.03	0.17*±0.02	0.16*±0.01	0.17*±0.00	0.38±0.02	0.56*±0.05	0.56*±0.01
**17**	α-Phellandren-8-ol	1148	0.26±0.02	0.18*±0.01	0.22±0.02	0.30±0.02	0.27±0.01	0.27±0.00	0.28±0.01	0.27±0.01	0.36*±0.03	0.38*±0.01
**18**	Borneol	1151	0.17±0.02	0.13±0.02	0.12±0.03	0.16±0.00	0.16±0.01	0.15±0.01	0.16±0.02	0.18±0.01	0.28*±0.00	0.28*±0.02
**19**	Terpinen-4-ol	1165	4.23±0.13	2.51*±0.10	2.83*±0.16	3.13*±0.23	3.10*±0.17	3.15*±0.11	3.09*±0.21	2.93*±0.15	3.94±0.22	3.92±0.16
**20**	Myrtenal	1172	0.15±0.02	0.12±0.04	0.11±0.02	0.15±0.01	0.15±0.00	0.14±0.01	0.16±0.00	0.13±0.02	0.19*±0.01	0.22*±0.02
**21**	α-Terpineol	1174	0.73±0.07	0.55*±0.02	0.57*±0.04	0.64±0.03	0.62±0.06	0.62±0.04	0.62±0.00	0.55*±0.03	0.81±0.05	0.75±0.03
**22**	Verbenone	1182	0.36±0.02	0.21*±0.01	0.30*±0.03	0.38±0.02	0.32±0.02	0.34±0.01	0.29*±0.01	0.34±0.00	0.58*±0.05	0.52*±0.03
**23**	Citronellol	1211	0.24±0.02	0.05*±0.00	0.11*±0.01	0.05*±0.03	0.08*±0.02	0.09*±0.01	0.08*±0.00	0.27±0.01	0.37*±0.03	0.41*±0.01
**24**	Citronellic acid	1244	0.24±0.02	0.20±0.02	0.18*±0.01	0.21±0.01	0.19±0.02	0.20±0.02	0.19*±0.02	0.21±0.01	0.34*±0.02	0.30*±0.03
**25**	Bornyl acetate	1271	0.40±0.04	0.27*±0.01	0.26*±0.00	0.29*±0.01	0.31*±0.03	0.30*±0.01	0.30*±0.00	0.29*±0.01	0.46±0.04	0.45±0.03
**26**	Carvacrol	1275	0.10±0.01	0.07*±0.00	0.07*±0.00	0.08±0.01	0.09±0.01	0.07±0.02	0.08±0.01	0.09±0.00	0.15*±0.01	0.14*±0.01
**27**	α-Terpinyl acetate	1334	0.32±0.03	0.68*±0.02	0.55*±0.05	0.48*±0.01	0.45*±0.04	0.39±0.04	0.47*±0.03	0.23*±0.01	0.38±0.03	0.29±0.02
**28**	α-Cubebene	1352	0.79±0.06	0.64*±0.01	0.69±0.04	0.73±0.03	0.81±0.05	0.79±0.01	0.80±0.02	0.77±0.04	1.14*±0.10	1.14*±0.09
**29**	α-Copaene	1379	0.70±0.06	0.53*±0.07	0.56*±0.01	0.64±0.03	0.71±0.02	0.69±0.02	0.68±0.03	0.68±0.05	0.97*±0.03	0.98*±0.05
**30**	β-Elemene	1389	1.34±0.10	1.06*±0.05	1.00*±0.03	1.12±0.12	1.32±0.13	1.24±0.06	1.24±0.05	1.13±0.11	1.59*±0.08	1.62*±0.06
**31**	α-Gurjunene	1402	0.29±0.02	0.24*±0.02	0.24*±0.01	0.27±0.02	0.34*±0.02	0.30±0.01	0.30±0.00	0.31±0.02	0.43*±0.03	0.44*±0.02
**32**	(*E*)-Caryophyllene	1422	4.03±0.34	2.92*±0.10	3.29*±0.04	3.51±0.25	3.90±0.15	3.81±0.12	3.72±0.19	3.55±0.21	5.53*±0.06	5.39*±0.14
**33**	γ-Elemene	1431	1.70±0.15	1.24*±0.09	1.40±0.15	1.58±0.04	1.79±0.10	1.67±0.06	1.54±0.13	0.84*±0.01	1.13*±0.04	1.38*±0.11
**34**	(*E*)-β-Farnesene	1449	0.81±0.06	0.62*±0.04	0.67*±0.02	0.74±0.05	0.82±0.01	0.78±0.04	0.81±0.00	0.84±0.07	1.16*±0.09	1.20*±0.07
**35**	α-Humulene	1455	2.75±0.19	1.97*±0.13	2.26*±0.15	2.42±0.17	2.67±0.22	2.56±0.14	2.53±0.19	2.51±0.20	3.94*±0.26	3.83*±0.14
**36**	γ-Muurolene	1474	0.51±0.05	0.41*±0.04	0.45±0.04	0.50±0.01	0.57±0.02	0.51±0.02	0.55±0.00	0.52±0.01	0.85*±0.02	0.85*±0.05
**37**	Germacrene D	1481	5.01±0.29	3.64*±0.15	3.92±0.13	4.40*±0.20	4.82±0.32	4.51±0.39	4.57±0.27	5.43±0.43	6.89*±0.25	7.39*±0.38
**38**	β-Selinene	1485	0.48±0.04	0.39*±0.02	0.39*±0.00	0.43±0.03	0.49±0.02	0.48±0.04	0.48±0.02	0.50±0.01	0.79*±0.03	0.79*±0.05
**39**	β-Cubebene	1489	0.26±0.02	0.19*±0.00	0.22±0.02	0.24±0.01	0.30±0.02	0.26±0.01	0.28±0.00	0.28±0.01	0.42*±0.01	0.43*±0.02
**40**	δ-Cadinene	1518	1.98±0.15	1.20*±0.16	1.47*±0.10	1.80±0.09	1.83±0.10	1.81±0.08	1.82±0.12	2.04±0.18	3.01*±0.17	3.00*±0.23
**41**	(*E*)-Nerolidol	1550	0.20±0.02	0.18±0.01	0.19±0.01	0.24±0.02	0.24±0.02	0.23±0.01	0.25*±0.02	0.27*±0.01	0.44*±0.13	0.44*±0.23
**42**	Caryophyllene epoxide	1574	1.06±0.08	1.03±0.06	0.93±0.07	0.98±0.03	0.98±0.07	0.96±0.09	0.97±0.06	1.11±0.10	2.01*±0.16	1.94*±0.12
**43**	Cedrol	1598	0.54±0.03	0.49±0.04	0.48±0.03	0.50±0.04	0.50±0.03	0.48±0.03	0.49±0.02	0.63*±0.05	1.13*±0.06	1.11*±0.03
**44**	Cubenol	1619	0.22±0.05	0.20±0.02	0.22±0.00	0.27±0.01	0.28±0.02	0.28±0.01	0.29±0.03	0.30*±0.02	0.54*±0.03	0.53*±0.03
**45**	α-Cadinol	1642	0.80±0.05	0.51*±0.03	0.75±0.07	0.88±0.04	0.84±0.05	0.74±0.03	0.77±0.04	0.93*±0.04	1.73*±0.08	1.65*±0.10
	**Total**		**93.76±6.9**	**92.69±3.1**	**92.28±5.3**	**90.78±4.07**	**90.10±3.18**	**90.95±2.71**	**89.93±2.39**	**89.12±3.61**	**83.14±3.16**	**82.91±4.13**

^a^Retention index (RI) is an average of all RIs in analysed samples. The results obtained were expressed as mean ± SD with n = 3 according to One-Way ANOVA.

*Values with superscript are significantly different compared to control sample at P < 0.05.

### Antioxidant Activity

The total phenolic content (TPC) is an important indicator of antioxidant capacity of plant extracts. The Folin–Ciocalteau method measures the level of total phenolic compounds occurred in natural products such as spices based on oxidation/reduction mechanisms [6]. The total phenolic content was found to be about 2-fold higher in sample 100/30 (15.47 mg CE/g of extract) than in the control sample (9.81 mg CE/g of extract) (Table 4). Comparing the results before and after ozone treatment, the phenolic content appeared significantly different. During short contact time, higher values of TPC were reported, 15.47 mg CE/g of extract and 12.91 mg CE/g of extract, for samples 100/30 and 130/30, respectively. Whereas samples 100/60, 130/60, 100/90, and 160/90 exhibited the lowest amount of phenolics. The results for juniper berries not treated with ozone correspond to those obtained by Orhan et al. (2011) [41] (11.92 ± 6.71 mg GAE/g of extract). As it was reported in our previous study, the degradation mechanism of polyphenols with ozone is still not well understood [16]. However, Diao et al. (2014) [42] proposed an ozonolysis pathways of nine polyphenols. In the mentioned study, it was shown that ozone (50.0 g O_3_/m^3^) at a flow rate of 5 L/min can destroy polyphenols during a 12-hour-treatment. Although our results demonstrate significant differences at P < 0.05 level in samples being treated with ozone, it does not indicate such great losses. This can be due to a different ozone treatment setup (shorter contact times, etc.). The most important is a good contact between plant material and ozone. The obtained results show that longer time of ozone treatment causes degradation of phenolic compounds. The time contact with ozone influences on phenolic content and it is due to a cleavage of glycosidic linkages with sugars or an oxidation of polyphenols to a carbonyl group [42].

**Table 4:** Determination of total polyphenol content (TPC) of methanolic extract from juniper (*J*. *communis* (L.)) berries after ozone treatments (mg CE/g of extract).

Ozone treatment	TPC (mg CE/g of extract)
**control**	9.81 ± 0.10^e^
**100/30**	15.47 ± 0.13^g^
**130/30**	12.91 ± 0.37^f^
**160/30**	8.10 ± 0.15^c^
**100/60**	5.96 ± 0.13^b^
**130/60**	6.22 ± 0.08^b^
**160/60**	9.07 ± 0.06^d^
**100/90**	6.16 ± 0.02^b^
**130/90**	8.77 ± 0.12^d^
**160/90**	5.18 ± 0.03^a^

The results obtained were expressed as mean ± SD with n = 3 according to One-Way ANOVA. Different letters (a-g) designate statistically significant differences between different ozone doses and times at P < 0.05. Total phenolic content expressed as mg of catechin equivalent (CE)/g of extract.

The content of polyphenols is closely correlated to the antioxidant capacity. Natural antioxidants have a wide mechanism of action. Accordingly, a single method of antioxidant activity is incapable of comprehending the antioxidant profile, thus different assays of antioxidant activity should be used [3, 5, 6, 43]. Therefore, in the present study the methanolic extracts and essential oils were examined for their free radical scavenging capacity towards the DPPH, FRAP, and BCB method. Above assays present different mechanisms of the determination of antioxidant capacity. The DPPH method measures the ability of the extract to donate a hydrogen to radical. The ferric reducing antioxidant power (FRAP) evaluates the capacity of the extract to reduce Fe^3+^ to Fe^2+^. The β-carotene bleaching test (BCB) measures the inhibition of coupled autooxidation of linoleic acid and β-carotene [5, 6, 43].

In the DPPH test the extract exhibited similar radical scavenging capacity before and after ozone treatment varied from 4.92 mg TE/g of sample to 5.02 mg TE/g of sample, respectively (Table 5). The IC_50_ value was found to be 7.63 μg/L before, and 6.86–13.68 μg/L after ozone treatment. Our results are in disagreement with those reported by Chaouche et al. (2015) [5], whereas extracts of *J*. *oxycedrus* showed lower scavenging activity (IC_50_ of 1.1 μg/mL). Furthermore, the total antioxidant capacity for essential oils before and after ozone treatment were slight different. The EOs are good scavengers of free radicals. The results present better free radical scavenging activity in samples during 90-min-treatment with ozone (2.32; 2.32; 1.27 mg/L) compared to the control sample (3.14 mg/L).

**Table 5:** Antioxidant activity (DPPH, FRAP, β-carotene inhibition) of methanolic extracts and essential oils from juniper (*J*. *communis* (L.)) berries after ozone treatments.

		DPPH (mg TE/g of sample)	IC_50/DPPH_ (μg/L)	FRAP (mM FeSO_4_ × 7 H_2_O)	β-carotene inhibition (%)
	Control	4.92 ± 0.03^a^	7.63 ± 0.02^b^	8.84 ± 0.43^d, e^	24.36 ± 1.07^a^
	100/30	4.96 ± 0.01^a^	7.74 ± 0.01^d^	10.70 ± 0.53^g^	27.04 ± 1.95^a^
	130/30	4.89 ± 0.02^a^	6.86 ± 0.00^a^	8.82 ± 0.22^e^	27.02 ± 1.31^a^
	160/30	5.02 ± 0.01^a^	8.11 ± 0.05^f^	9.39 ± 0.10^f^	27.84 ± 1.11^a, b^
**methanolic extract**	100/60	4.96 ± 0.04^a^	13.68 ±0.02^i^	5.87 ± 0.11^a^	31.48 ± 0.06^e^
	130/60	4.79 ± 0.28^a^	9.39± 0.08^h^	7.42 ± 0.05^c^	29.11 ± 0.13^b, c^
	160/60	4.76 ± 0.21^a^	7.79 ± 0.03^d^	8.89 ± 0.02^e^	26.33 ± 1.02^a, b^
	100/90	5.02 ± 0.00^a^	7.70 ± 0.01^c^	8.83 ± 0.06^e^	30.63 ± 0.15^d^
	130/90	5.00 ± 0.04^a^	8.68 ± 0.09^g^	8.39 ± 0.05^d^	27.68 ± 1.09^a, b^
	160/90	5.01 ± 0.01^a^	7.85 ± 0.03^e^	6.90 ± 0.04^b^	27.48 ± 1.10^a, b^
	Control	0.46 ± 0.01^c^	3.14 ± 0.03^f^	0.99 ± 0.00^e^	2.39 ± 0.03^d^
	100/30	0.36 ± 0.02^a^	3.85 ± 0.04^g^	0.91 ± 0.02^d^	2.28 ± 0.06^c^
	130/30	0.34 ± 0.00^a^	4.25 ± 0.02^h^	0.91 ± 0.11^d^	2.25 ± 0.05^c^
	160/30	0.38 ± 0.00^b^	2.20 ± 0.03^c, d^	1.11 ± 0.02^e^	2.21 ± 0.12^c^
**essential oil**	100/60	0.37 ± 0.01^a^	2.19 ± 0.08^c^	0.47 ± 0.02^a^	1.19 ± 0.08^a^
	130/60	0.54 ± 0.00^d^	1.47 ± 0.02^b^	0.52 ± 0.02^b^	1.28 ± 0.07^a^
	160/60	0.35 ± 0.00^a^	2.13 ± 0.00^c^	0.56 ± 0.01^c^	1.23 ± 0.05^a^
	100/90	0.36 ± 0.01^a^	2.32 ± 0.01^e^	0.67 ± 0.11^c^	1.96 ± 0.08^b^
	130/90	0.66 ± 0.01^f^	2.32 ± 0.05^e^	0.82 ± 0.15^c, d^	2.30 ± 0.09^c^
	160/90	0.61 ± 0.01^e^	1.27 ± 0.08^a^	0.87 ± 0.16^c, d^	2.34 ± 0.13^c, d^

The results obtained were expressed as mean ± SD with n = 3 according to One-Way ANOVA. Different letters (a-i) in columns designate statistically significant differences between different ozone doses and times at P < 0.05.

In the FRAP assay the extracts as well as essential oils from berries not treated with ozone showed a good ferric reducing power: 8.84, and 0.99 mM FeSO_4_ x 7 H_2_O, respectively (Table 5). The sample 100/30 exhibited the highest reducing power 10.70 mM FeSO_4_ x 7 H_2_O followed by samples 160/30 and 130/30 (9.39 and 8.82 mM FeSO_4_ x 7 H_2_O, respectively). This study indicated that EOs (samples 100/60, 130/60, 160/60) were significantly affected by ozone, about 50% lower FRAP power is observed compared to the control sample. The reducing power of extracts may be caused by the action of the hydroxyl group of the phenolic compounds which might act as electron donors [44].

In the β-carotene bleaching test the highest bleaching activities were demonstrated for samples 100/60, 130/60, and 100/90, with 31.48; 29.11; and 30.63% inhibition of β-carotene, respectively (Table 5). It can be explained by a better transfer of the hydrogen atom from phenolic compounds to the free radical, and therefore inhibiting bleaching of β-carotene [44]. Extracts obtained from juniper berries after ozone treatment possessed significantly different bleaching activity in comparison to the control sample (24.36 ± 1.07%).

A decrease of antioxidant activity in all presented assays is probably caused by attacking of carbon-carbon double bonds by ozone, which gives rise to a new dicarbonyl compound. Additionally, ozone–phenol reactions are faster than ozone–benzene reactions, but they are influenced by the numbers and positions of -OH groups present on the compound structure [16].

### Phenolic Profile

The identification of phenolics in MeOH extracts from ozonated and not ozonated juniper berries was carried out using the LC-MS technique in negative mode. Overall data concerning the quality of identified compounds. Using this procedure, 21 and 31 phenolic compounds before and after ozone treatment, respectively, were characterized. Molecule identification was based on the UV spectrum, the exact mass and MS/MS fragmentation pattern summarized in Table 6. According to the results, there were no significant differences in quality of the juniper extracts from berries during ozone treatment. Compounds 1–2, and 13 displayed similar λ_max_ and MS/MS which allowed to identify them as phenolic acids and their probably derivatives such as quinic acid and gallic acid [43, 44, 45]. Compounds 3, 5, 7, 8 19 in not ozonated samples and 3, 5, 7, 8, 14, 19 in samples after ozone treatments presented [M–H]^-^ fragments, which firstly may indicate that they could be close to the anthocyanins, however λ_max_ does not allow to identify them as such class of flavonoids [16]. Therefore, additional structural analyses in NMR would be necessary to correctly identify them. Compounds 4 and 10 were not detected in control sample, but in ozonated sample (160/30) having an expected exact mass of 159 which corresponds to the coumarins. The major [M–H]^-^ fragments at 114, 115, 129 and 141 are characteristic to umbelliferone. Compound 6 exhibited a [M–H]^-^ with a m/z of 159. Its fragmentation pattern in negative mode matched that of protocatechuic acid [43, 44]. Compound 9 exhibited a [M–H]^-^ m/z at 395, thus it was assigned as a stilbene derivative [16]. Compound 11 has an exact mass at 289, and characteristic MS/MS fragments at 205 and 245. This together with λ_max_ allowed to identify this compound as catechin [43, 45]. Compounds 12, 14, 16 and 21 were not detected in the control samples. The same compounds (12, 16, 21) occurred in samples after ozone treatment and exhibited exact mass at 403, 345, and 209, respectively. The λ_max_ and MS/MS fragmentation allows to identify them as caffeic acid derivatives and 3, 4-dimethoxycinnamic acid, respectively. Compound 15, detected only in the control sample, exhibited a [M–H]^-^ parent ion at m/z 345 corresponding to a coumaric acid derivative [45]. Compounds 17 and 18 displayed similar MS/MS fragments, namely 317 which indicates myricetin, 449—myricetin arabinoside, and 479—myricetin 3-O-galactoside. This together with λ_max_ allowed to identify this compound as myricetin derivatives. Compounds 20, 22–24, and 27 for control samples and compounds 20, 22–24 and 27 for samples after ozone treatments exhibited closely related λ_max_ and MS/MS fragmentation patterns indicating that they belong to the same structural family of the glycosylated forms of flavonols [45]. Compounds 25 and 26 displayed similar λ_max_, however different MS/MS fragments, while they were not detected before ozone treatment. Due to their close relation they were assigned as quercetin and quercetin hexose, respectively [5, 45]. Compounds 28–33 belong to the class of flavonoids such as flavones, λ_max_ and MS/MS fragmentation patterns are characteristic for kaempferol, luteolin, chrysoeriol (ozonated samples), apigenin, amentoflavone, and cupressoflavone [6, 43, 44, 45]. Compound 34, 36, and 37 occurred only in samples after ozone treatment and had a characteristic exact mass, MS/MS fragments and λ_max_ for phenolic acids such as hydroxybenzoic and p-coumaroyloquinic acids [44, 45]. Compound 35 exhibited a [M–H]^-^ parent ion at m/z 225 allowing to identify as sinapic acid [16].

**Table 6:** LC-MS analysis of phenolics identified in methanolic extracts from juniper (*J*. *communis* (L.)) berries after ozone treatments.

Control						After ozone treatments					
Peak	RT^a^ (min)	Λ_max_ (nm)	[M–H]^-^	MS-MS [M–H]^-^	Proposed molecule	RT^b^ (min)	λ_max_ (nm)	[M–H]^-^	MS-MS [M–H]^-^	Proposed molecule	Parameters of ozone treatment
**1**	3.35	235, 284	191	111, 173	Quinic acid	3.36	235, 276	191	111, 173	Quinic acid	A, B, C, D, E, F, G, H, I
**2**	4.30	237, 276	205	111, 125, 173, 187	Gallic acid derivative	4.26	238, 276	205	111, 125, 173	Gallic acid derivative	A, B, D, E, F, G, H, I
**3**	5.68	237	405	359	Unknown compound	5.67	239	439	265	Unknown compound	B
**4**	5.91	nd				5.91	235	159	114, 115, 129, 141	Umbelliferone	C
**5**	6.92	254	375	139, 201, 345, 357	Unknown compound	6.80	239	375	139, 201, 217, 357	Unknown compound	A, B
**6**	7.36	260, 294	153	109	Protocatechuic acid	7.49	235, 259, 294	153	109	Protocatechuic acid	A, B, C, D, E, F, G, H, I
**7**	8.96	238	447	401	Unknown compound	8.96	nd				
**8**	9.31	238, 270	443	161, 219, 237, 281, 425	Unknown compound	9.43	235, 276	443	161, 219, 237, 425	Unknown compound	B, C, D, E, F, G, H, I
**9**	10.18	240, 253	395	349	Stilbene derivative	10.18	245	395	349	Stilbene derivative	A, B, D, E, F, G, H, I
**10**	10.33	nd				10.33	235	159	115, 116, 132, 141	Umbelliferone	C
**11**	10.70	238, 280	289	205, 245	Catechin	10.72	239, 280	289	205, 245	Catechin	B, E, F
**12**	10.83	nd				10.83	235, 280, 309	403	179, 357	Caffeic acid derivative	C, E, F, G, H, I
**13**	11.73	238, 273, 351	641	479	Gallic acid derivative	11.74	238, 271, 352	641	317, 479	Gallic acid derivative	D, F
**14**	11.80	nd				11.80	235, 270	431	385	Unknown compound	A, B, C, D, E, F, G, H, I
**15**	12.98	238, 275, 344, 396	345	161, 301	Coumaric acid derivative	12.98	nd				
**16**	13.03	nd				13.03	235, 274	345	161, 301	Caffeic acid derivative	A, B, C, D, E, F, G, H, I
**17**	14.71	238, 269, 354	611	317, 449, 479	Myricetin derivatives	14.83	235, 267, 330	611	317, 449, 479	Myricetin derivatives	A, B, C, D, E, F, G, H, I
**18**	16.57	237, 269, 352, 396	641	317, 479	Myricetin derivatives	16.68	235	641	317, 479	Myricetin derivatives	A, B, C, D, E, F, G, H, I
**19**	18.36	237, 271, 348	655	331, 493	Unknown compound	18.38	235, 268, 301	655	331, 493	Unknown compound	A, B, C, D, E, F, G, H, I
**20**	20.93	275, 343	463	301	Quercetin 3-O-galactoside	21.11	236, 276, 344, 397	463	301	Quercetin 3-O-galactoside	A, B, C, D, E, F, G, H, I
**21**	22.75	nd				22.75	235, 268, 342	209	79, 153	3,4-Dimethoxycinnamic acid	F
**22**	25.84	237, 276, 341, 396	433	301	Quercetin 3-O-arabinofuranoside	25.93	235, 276, 330, 342	433	301	Quercetin 3-O-arabinofuranoside	A, B, C, D, E, F, G, H, I
**23**	27.29	238, 277, 305, 327	447	285, 301	Luteolin-O-galactoside	27.38	235, 277, 306, 329	447	285	Luteolin-O-galactoside	A, B, C, D, E, F, G, H, I
**24**	28.32	271, 297, 341, 362	433	301	Quercetin 3- O-arabinopyranoside	28.47	237, 276, 344, 396	433	301	Quercetin 3-O-arabinopyranoside	A, B, C, D, E, F, G, H, I
**25**	28.95	nd				28.95	235, 279, 341, 396	301	165, 201, 229, 255, 257	Quercetin	A, C, D, E, F, G, H, I
**26**	30.33	nd				30.33	235, 274	461	283, 446	Quercetin hexose	A, C, D, E, F, G, H, I
**27**	33.71	242, 275, 304, 328	417	285	Kaempferol pentoside	33.78	237, 276, 306, 328	417	285	Kaempferol pentoside	A, B, C, D, E, F, G, H, I
**28**	34.44	239, 279, 303, 325, 396	285	213, 341, 257	Kaempferol	34.58	235, 280, 304, 328	285	192, 213, 241, 267	Kaempferol	A, B, C, D, E, F, G, H, I
**29**	38.68	237, 270, 345, 397	285	175, 199, 217, 241, 243, 267	Luteolin	38.96	235, 310, 345	285	175, 199, 217, 241, 243	Luteolin	A, B, C, D, E, F, G, H, I
**30**	44.57	nd				44.57	235	299	284	Chrysoeriol	A, B, C, D, E, F, G, H, I
**31**	45.23	237, 269, 337, 397	269	149, 181, 201, 225, 227, 251	Apigenin	45.37	236, 268	269	149, 201, 225	Apigenin	A, B, C, D, E, F, G, H, I
**32**	50.52	236, 273, 329, 397	537	375, 443	Amentoflavone	50.45	236, 274, 329	537	375, 443	Amentoflavone	A, B, C, D, E, G, H, I
**33**	55.68	236, 269, 334, 397	537	375, 417, 443	Cupressoflavone	55.57	236	537	375, 417, 443	Cupressoflavone	A, B, C, D, E, F, G, H, I
**34**	55.38	nd				58.38	236	130	71, 85, 87, 102, 113	Hydroxybenzoic acid	C, D, E, F, G
**35**	58.53	236	225	141, 156, 181, 207	Sinapic acid	61.23	239, 292	225	141, 156, 181, 197	Sinapic acid	A
**36**	61.27	nd				61.27	236, 293	130	59, 102, 103, 113	Hydroxybenzoic acid	C, D, E, F, G
**37**	61.37	nd				61.37	237, 291	337	257, 291, 319	p-Coumaroylquinic acid	B

^a, b^ RT (Retention time) is an average of all RTs in analysed samples.

A– 100/30; B– 130/30; C– 160/30; D– 100/60; E– 130/60; F– 160/60; G– 100/90; H– 130/90; I– 160/90

nd–not detected

No previous work has been found on the identification of phenolic compounds in the berries of *J*. *communis* (L.) during ozone treatment. The berries’ phenolic composition does not differ much from that after ozone treatment. Indeed, berries treated with ozone contain more phenolics such as coumarins (umbelliferone), flavones or its derivatives: chrysoeriol, quercetin and quercetin hexose, as well as phenolic acids, namely hydroxybenzoic and p-coumaroyloquinic acids.

## Conclusion

The results of the present study revealed that berries of *Juniperus communis* (L.) treated as well as not treated with ozone are a rich source of phenolics. The various parameters (ozone concentration and contact time) in a dynamic bed do not significantly affect the quality of the berries, and even indicate better antioxidant activity after ozone treatment. However, ozone treatment was not significantly effective in the reduction of bacteria and fungi. Therefore, the next study will refer to determine the decay kinetics of selected bacteria and fungi during ozone treatment in a dynamic bed. Nevertheless, this paper established that ozone treatment possesses a promising potential as a decontamination method, which allows to obtain a product having sensory characteristics unchanged.

## Supporting Information

S1 Fig Ozone treatment system in dynamic bed for gaseous phase used for laboratory purposes; 1-oxygen bottle, 2-ozone generator, 3-ozone analyzer, 4- surplus gas elimination unit, 5-inlet of ozone, 6-outlet of ozone, 7-reactor, 8- control system with jolting and rotating mechanism, 9- supply and disposal of plant material treated with ozone. (DOCX)

S1 Table Occurrence of microbial contamination in common juniper (*J*. *communis* (L.)) berries after ozone treatments. (DOCX)

S2 Table Detection of the most dominant bacterial species of juniper (*J*. *communis* (L.)) berries after ozone treatments. (DOCX)

S3 Table Comparison of the composition of essential oil of juniper (*J*. *communis* (L.)) berries obtained after ozone treatments. (DOCX)

S4 Table Determination of total polyphenol content (TPC) of methanolic extract from juniper (*J*. *communis* (L.)) berries after ozone treatments (mg CE/g of extract). (DOCX)

S5 Table Antioxidant activity (DPPH, FRAP, β-carotene inhibition) of methanolic extracts and essential oils from juniper (*J*. *communis* (L.)) berries after ozone treatments. (DOCX)

S6 Table LC-MS analysis of phenolics identified in methanolic extracts from juniper (*J*. *communis* (L.)) berries after ozone treatments. (DOCX)
