# Supplementary material for: The Impact of Ozone Treatment in Dynamic Bed Parameters on Changes in Biologically Active Substances of Juniper Berries
Source: PLoS One. 2015 Dec 14;10(12):e0144855. doi: 10.1371/journal.pone.0144855 (PMC4678966; doi:10.1371/journal.pone.0144855)
Supplement: S1 Fig — (DOCX) [file pone.0144855.s001.docx]

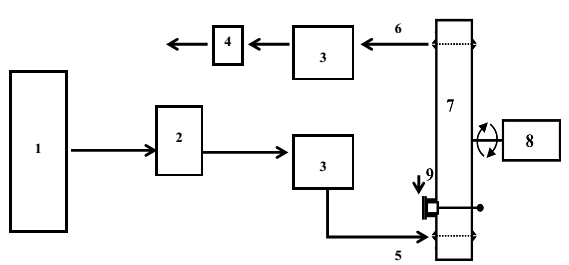


**S Fig. Ozone treatment system in dynamic bed for gaseous phase used for laboratory purposes; 1-oxygen bottle, 2-ozone generator, 3-ozone analyzer, 4- surplus gas elimination unit, 5-inlet of ozone, 6-outlet of ozone, 7-reactor, 8- control system with jolting and rotating mechanism, 9- supply and disposal of plant material treated with ozone**
