# Supplementary material for: The Impact of Ozone Treatment in Dynamic Bed Parameters on Changes in Biologically Active Substances of Juniper Berries
Source: PLoS One. 2015 Dec 14;10(12):e0144855. doi: 10.1371/journal.pone.0144855 (PMC4678966; doi:10.1371/journal.pone.0144855)
Supplement: S1 Table — (DOCX) [file pone.0144855.s002.docx]

**S1 Table. Occurrence of microbial contamination in common juniper (*J. communis* (L.)) berries after ozone treatments.**

| Ozone treatment | Total mesophilic bacteria count | Total fungal count | *Enterobacteriaceae* count |
| --- | --- | --- | --- |
| control | 5.2 ± 0.05^d^ | 5.2 ± 0.06^c^ | < 1* |
| 100/30 | 4.6 ± 0.12^c^ | 4.7 ± 0.07^b^ | < 1* |
| 130/30 | 3.8 ± 0.01^a^ | 4.2 ± 0.11^a^ | < 1* |
| 160/30 | 4.2 ± 0.03^b^ | 4.4 ± 0.07^a^ | < 1* |
| control | 5.1 ± 0.10^a^ | 5.5 ± 0.04^b^ | < 1* |
| 100/60 | 5.5 ± 0.04^b^ | 6.0 ± 0.19^d^ | < 1* |
| 130/60 | 5.4 ± 0.03^b^ | 5.3 ± 0.10^c^ | < 1* |
| 160/60 | 5.0 ± 0.03^a^ | 4.9 ± 0.14^a^ | < 1* |
| control | 6.1 ± 0.10^c^ | 5.8 ± 0.02^c^ | < 1* |
| 100/90 | 5.3 ± 0.03^b^ | 5.2 ± 0.02^b^ | < 1* |
| 130/90 | 4.5 ± 0.08^a^ | 4.2 ± 0.11^a^ | < 1* |
| 160/90 | 4.6 ± 0.02^a^ | 4.4 ± 0.12^a^ | < 1* |

All results are given as log (cfu g^-1^). The results obtained were expressed as mean ± SD with n=3 according to One-Way ANOVA. Different letters (a-d) in columns designate statistically significant differences between different ozone doses at the same time (P < 0.05).

^*^not detected at the level 10 cfu per 1 gram
