# Supplementary material for: The Impact of Ozone Treatment in Dynamic Bed Parameters on Changes in Biologically Active Substances of Juniper Berries
Source: PLoS One. 2015 Dec 14;10(12):e0144855. doi: 10.1371/journal.pone.0144855 (PMC4678966; doi:10.1371/journal.pone.0144855)
Supplement: S2 Table — (DOCX) [file pone.0144855.s003.docx]

**S2 Table. Detection of the most dominant bacterial species of juniper (*J. communis* (L.)) berries after ozone treatments.**

| Ozone treatment | Percentage distribution of species | | |
| --- | --- | --- | --- |
|  | *Bacillus subtilis* | *Bacillus pumilus* | *Bacillus cereus* |
| control | 70 | 10 | 20 |
| 100/30 | 70 | 15 | 15 |
| 130/30 | 60 | 20 | 20 |
| 160/30 | 60 | 15 | 25 |
| 100/60 | 65 | 20 | 15 |
| 130/60 | 65 | 25 | 5 |
| 160/60 | 75 | 15 | 15 |
| 100/90 | 75 | 20 | 5 |
| 130/90 | 65 | 20 | 10 |
| 160/90 | 65 | 25 | 5 |
