# Supplementary material for: The Impact of Ozone Treatment in Dynamic Bed Parameters on Changes in Biologically Active Substances of Juniper Berries
Source: PLoS One. 2015 Dec 14;10(12):e0144855. doi: 10.1371/journal.pone.0144855 (PMC4678966; doi:10.1371/journal.pone.0144855)
Supplement: S3 Table — (DOCX) [file pone.0144855.s004.docx]

**S3 Table. Comparison of the composition of essential oil of juniper (*J. communis* (L.)) berries obtained after ozone treatments.**

|  | | | Control | After ozone treatment | | | | | | | | |
| --- | --- | --- | --- | --- | --- | --- | --- | --- | --- | --- | --- | --- |
|  |  |  |  | **100/30** | **130/30** | **160/30** | **100/60** | **130/60** | **160/60** | **100/90** | **130/90** | **160/90** |
| No | **Compounds** | **RI^a^** | **%** | | | | | | | | | |
| 1 | α-Thujene | 926 | 0.64±0.09 | 0.78±0.11 | 0.80±0.08 | 0.73±0.09 | 0.63±0.12 | 0.68±0.05 | 0.67±0.11 | 0.72±0.06 | 0.41*±0.02 | 0.37*±0.08 |
| 2 | α-Pinene | 936 | 32.65±2.23 | 41.93*±0.79 | 38.70*±2.85 | 35.65±1.56 | 32.42±0.16 | 34.26±0.45 | 33.51±0.22 | 32.70±0.46 | 19.88*±0.37 | 17.48*±0.90 |
| 3 | Camphene | 946 | 0.31±0.08 | 0.35±0.02 | 0.35±0.00 | 0.33±0.02 | 0.31±0.00 | 0.27±0.01 | 0.32±0.01 | 0.31±0.02 | 0.23±0.01 | 0.20*±0.02 |
| 4 | Sabinene | 969 | 3.86±0.33 | 4.64*±0.10 | 4.38*±0.08 | 4.15±0.05 | 4.04±0.02 | 4.17±0.06 | 4.04±0.03 | 4.28*±0.12 | 2.73*±0.07 | 2.85*±0.11 |
| 5 | β-Pinene | 973 | 2.53±0.08 | 2.49±0.23 | 2.46±0.18 | 2.41±0.10 | 2.32±0.13 | 2.28*±0.11 | 2.28*±0.09 | 2.40±0.14 | 1.81*±0.03 | 1.81*±0.06 |
| 6 | β-Myrcene | 987 | 13.74±1.26 | 12.98±0.19 | 13.28±0.23 | 12.19*±0.15 | 12.97±0.57 | 13.56±0.08 | 13.14±0.04 | 12.93±0.63 | 8.90*±0.17 | 9.58*±0.10 |
| 7 | α-Terpinene | 1009 | 0.46±0.04 | 0.30*±0.02 | 0.42±0.02 | 0.43±0.02 | 0.38±0.04 | 0.34*±0.03 | 0.39±0.03 | 0.34*±0.03 | 0.29*±0.01 | 0.34*±0.00 |
| 8 | β-Cymene | 1013 | 0.66±0.05 | 0.49*±0.03 | 0.56±0.05 | 0.60±0.03 | 0.56±0.05 | 0.55±0.06 | 0.58±0.04 | 0.52*±0.03 | 0.59±0.04 | 0.62±0.04 |
| 9 | Limonene | 1024 | 5.14±0.26 | 4.24*±0.19 | 4.62±0.39 | 4.51±0.40 | 5.09±0.13 | 4.71±0.27 | 4.72±0.16 | 4.23*±0.11 | 3.75*±0.09 | 4.56±0.28 |
| 10 | γ-Terpinene | 1052 | 0.83±0.07 | 0.55*±0.03 | 0.75±0.02 | 0.76±0.01 | 0.70±0.06 | 0.65*±0.05 | 0.70±0.07 | 0.60*±0.05 | 0.59*±0.03 | 0.70±0.06 |
| 11 | α-Terpinolene | 1081 | 0.74±0.06 | 0.56*±0.02 | 0.66±0.04 | 0.68±0.01 | 0.69±0.02 | 0.64±0.04 | 0.68±0.04 | 0.61±0.05 | 0.61±0.05 | 0.66±0.04 |
| 12 | Linalool | 1085 | 0.18±0.05 | 0.20±0.02 | 0.15±0.01 | 0.19±0.00 | 0.20±0.01 | 0.16±0.01 | 0.21±0.02 | 0.21±0.00 | 0.19±0.01 | 0.28±0.05 |
| 13 | α-Campholenal | 1103 | 0.34±0.06 | 0.25±0.05 | 0.24*±0.02 | 0.28±0.02 | 0.27±0.02 | 0.28±0.01 | 0.30±0.00 | 0.27±0.02 | 0.39±0.03 | 0.42±0.03 |
| 14 | *cis*-p-Menth-2-en-1-ol | 1107 | 0.13±0.01 | 0.09*±0.00 | 0.09*±0.01 | 0.12±0.01 | 0.11±0.02 | 0.11±0.01 | 0.10±0.02 | 0.11±0.01 | 0.14±0.02 | 0.15±0.01 |
| 15 | *trans*-Pinocarveol | 1124 | 0.45±0.04 | 0.26*±0.01 | 0.25*±0.02 | 0.30*±0.00 | 0.29*±0.05 | 0.31*±0.01 | 0.31*±0.01 | 0.38±0.03 | 0.51±0.04 | 0.56*±0.05 |
| 16 | *cis*-Verbenol | 1129 | 0.43±0.03 | 0.15*±0.00 | 0.12*±0.01 | 0.33±0.03 | 0.17*±0.02 | 0.16*±0.01 | 0.17*±0.00 | 0.38±0.02 | 0.56*±0.05 | 0.56*±0.01 |
| 17 | α-Phellandren-8-ol | 1148 | 0.26±0.02 | 0.18*±0.01 | 0.22±0.02 | 0.30±0.02 | 0.27±0.01 | 0.27±0.00 | 0.28±0.01 | 0.27±0.01 | 0.36*±0.03 | 0.38*±0.01 |
| 18 | Borneol | 1151 | 0.17±0.02 | 0.13±0.02 | 0.12±0.03 | 0.16±0.00 | 0.16±0.01 | 0.15±0.01 | 0.16±0.02 | 0.18±0.01 | 0.28*±0.00 | 0.28*±0.02 |
| 19 | Terpinen-4-ol | 1165 | 4.23±0.13 | 2.51*±0.10 | 2.83*±0.16 | 3.13*±0.23 | 3.10*±0.17 | 3.15*±0.11 | 3.09*±0.21 | 2.93*±0.15 | 3.94±0.22 | 3.92±0.16 |
| 20 | Myrtenal | 1172 | 0.15±0.02 | 0.12±0.04 | 0.11±0.02 | 0.15±0.01 | 0.15±0.00 | 0.14±0.01 | 0.16±0.00 | 0.13±0.02 | 0.19*±0.01 | 0.22*±0.02 |
| 21 | α-Terpineol | 1174 | 0.73±0.07 | 0.55*±0.02 | 0.57*±0.04 | 0.64±0.03 | 0.62±0.06 | 0.62±0.04 | 0.62±0.00 | 0.55*±0.03 | 0.81±0.05 | 0.75±0.03 |
| 22 | Verbenone | 1182 | 0.36±0.02 | 0.21*±0.01 | 0.30*±0.03 | 0.38±0.02 | 0.32±0.02 | 0.34±0.01 | 0.29*±0.01 | 0.34±0.00 | 0.58*±0.05 | 0.52*±0.03 |
| 23 | Citronellol | 1211 | 0.24±0.02 | 0.05*±0.00 | 0.11*±0.01 | 0.05*±0.03 | 0.08*±0.02 | 0.09*±0.01 | 0.08*±0.00 | 0.27±0.01 | 0.37*±0.03 | 0.41*±0.01 |
| 24 | Citronellic acid | 1244 | 0.24±0.02 | 0.20±0.02 | 0.18*±0.01 | 0.21±0.01 | 0.19±0.02 | 0.20±0.02 | 0.19*±0.02 | 0.21±0.01 | 0.34*±0.02 | 0.30*±0.03 |
| 25 | Bornyl acetate | 1271 | 0.40±0.04 | 0.27*±0.01 | 0.26*±0.00 | 0.29*±0.01 | 0.31*±0.03 | 0.30*±0.01 | 0.30*±0.00 | 0.29*±0.01 | 0.46±0.04 | 0.45±0.03 |
| 26 | Carvacrol | 1275 | 0.10±0.01 | 0.07*±0.00 | 0.07*±0.00 | 0.08±0.01 | 0.09±0.01 | 0.07±0.02 | 0.08±0.01 | 0.09±0.00 | 0.15*±0.01 | 0.14*±0.01 |
| 27 | α-Terpinyl acetate | 1334 | 0.32±0.03 | 0.68*±0.02 | 0.55*±0.05 | 0.48*±0.01 | 0.45*±0.04 | 0.39±0.04 | 0.47*±0.03 | 0.23*±0.01 | 0.38±0.03 | 0.29±0.02 |
| 28 | α-Cubebene | 1352 | 0.79±0.06 | 0.64*±0.01 | 0.69±0.04 | 0.73±0.03 | 0.81±0.05 | 0.79±0.01 | 0.80±0.02 | 0.77±0.04 | 1.14*±0.10 | 1.14*±0.09 |
| 29 | α-Copaene | 1379 | 0.70±0.06 | 0.53*±0.07 | 0.56*±0.01 | 0.64±0.03 | 0.71±0.02 | 0.69±0.02 | 0.68±0.03 | 0.68±0.05 | 0.97*±0.03 | 0.98*±0.05 |
| 30 | β-Elemene | 1389 | 1.34±0.10 | 1.06*±0.05 | 1.00*±0.03 | 1.12±0.12 | 1.32±0.13 | 1.24±0.06 | 1.24±0.05 | 1.13±0.11 | 1.59*±0.08 | 1.62*±0.06 |
| 31 | α-Gurjunene | 1402 | 0.29±0.02 | 0.24*±0.02 | 0.24*±0.01 | 0.27±0.02 | 0.34*±0.02 | 0.30±0.01 | 0.30±0.00 | 0.31±0.02 | 0.43*±0.03 | 0.44*±0.02 |
| 32 | (*E*)-Caryophyllene | 1422 | 4.03±0.34 | 2.92*±0.10 | 3.29*±0.04 | 3.51±0.25 | 3.90±0.15 | 3.81±0.12 | 3.72±0.19 | 3.55±0.21 | 5.53*±0.06 | 5.39*±0.14 |
| 33 | γ-Elemene | 1431 | 1.70±0.15 | 1.24*±0.09 | 1.40±0.15 | 1.58±0.04 | 1.79±0.10 | 1.67±0.06 | 1.54±0.13 | 0.84*±0.01 | 1.13*±0.04 | 1.38*±0.11 |
| 34 | (*E*)-β-Farnesene | 1449 | 0.81±0.06 | 0.62*±0.04 | 0.67*±0.02 | 0.74±0.05 | 0.82±0.01 | 0.78±0.04 | 0.81±0.00 | 0.84±0.07 | 1.16*±0.09 | 1.20*±0.07 |
| 35 | α-Humulene | 1455 | 2.75±0.19 | 1.97*±0.13 | 2.26*±0.15 | 2.42±0.17 | 2.67±0.22 | 2.56±0.14 | 2.53±0.19 | 2.51±0.20 | 3.94*±0.26 | 3.83*±0.14 |
| 36 | γ-Muurolene | 1474 | 0.51±0.05 | 0.41*±0.04 | 0.45±0.04 | 0.50±0.01 | 0.57±0.02 | 0.51±0.02 | 0.55±0.00 | 0.52±0.01 | 0.85*±0.02 | 0.85*±0.05 |
| 37 | Germacrene D | 1481 | 5.01±0.29 | 3.64*±0.15 | 3.92±0.13 | 4.40*±0.20 | 4.82±0.32 | 4.51±0.39 | 4.57±0.27 | 5.43±0.43 | 6.89*±0.25 | 7.39*±0.38 |
| 38 | β-Selinene | 1485 | 0.48±0.04 | 0.39*±0.02 | 0.39*±0.00 | 0.43±0.03 | 0.49±0.02 | 0.48±0.04 | 0.48±0.02 | 0.50±0.01 | 0.79*±0.03 | 0.79*±0.05 |
| 39 | β-Cubebene | 1489 | 0.26±0.02 | 0.19*±0.00 | 0.22±0.02 | 0.24±0.01 | 0.30±0.02 | 0.26±0.01 | 0.28±0.00 | 0.28±0.01 | 0.42*±0.01 | 0.43*±0.02 |
| 40 | δ-Cadinene | 1518 | 1.98±0.15 | 1.20*±0.16 | 1.47*±0.10 | 1.80±0.09 | 1.83±0.10 | 1.81±0.08 | 1.82±0.12 | 2.04±0.18 | 3.01*±0.17 | 3.00*±0.23 |
| 41 | (*E*)-Nerolidol | 1550 | 0.20±0.02 | 0.18±0.01 | 0.19±0.01 | 0.24±0.02 | 0.24±0.02 | 0.23±0.01 | 0.25*±0.02 | 0.27*±0.01 | 0.44*±0.13 | 0.44*±0.23 |
| 42 | Caryophyllene epoxide | 1574 | 1.06±0.08 | 1.03±0.06 | 0.93±0.07 | 0.98±0.03 | 0.98±0.07 | 0.96±0.09 | 0.97±0.06 | 1.11±0.10 | 2.01*±0.16 | 1.94*±0.12 |
| 43 | Cedrol | 1598 | 0.54±0.03 | 0.49±0.04 | 0.48±0.03 | 0.50±0.04 | 0.50±0.03 | 0.48±0.03 | 0.49±0.02 | 0.63*±0.05 | 1.13*±0.06 | 1.11*±0.03 |
| 44 | Cubenol | 1619 | 0.22±0.05 | 0.20±0.02 | 0.22±0.00 | 0.27±0.01 | 0.28±0.02 | 0.28±0.01 | 0.29±0.03 | 0.30*±0.02 | 0.54*±0.03 | 0.53*±0.03 |
| 45 | α-Cadinol | 1642 | 0.80±0.05 | 0.51*±0.03 | 0.75±0.07 | 0.88±0.04 | 0.84±0.05 | 0.74±0.03 | 0.77±0.04 | 0.93*±0.04 | 1.73*±0.08 | 1.65*±0.10 |
|  | **Total** |  | **93.76±6.9** | **92.69±3.1** | **92.28±5.3** | **90.78±4.07** | **90.10±3.18** | **90.95±2.71** | **89.93±2.39** | **89.12±3.61** | **83.14±3.16** | **82.91±4.13** |

^a^Retention index (RI) is an average of all RIs in analysed samples. The results obtained were expressed as mean ± SD with n=3 according to One-Way ANOVA. Values with superscript are significantly different compared to control sample at P < 0.05.
