# Supplementary material for: The Impact of Ozone Treatment in Dynamic Bed Parameters on Changes in Biologically Active Substances of Juniper Berries
Source: PLoS One. 2015 Dec 14;10(12):e0144855. doi: 10.1371/journal.pone.0144855 (PMC4678966; doi:10.1371/journal.pone.0144855)
Supplement: S4 Table — (DOCX) [file pone.0144855.s005.docx]

**S4 Table. Determination of total polyphenol content (TPC) of methanolic extract from juniper (*J. communis* (L.)) berries after ozone treatments (mg CE/g of extract).**

| Ozone treatment | TPC (mg CE/g of extract) |
| --- | --- |
| control | 9.81 ± 0.10^e^ |
| 100/30 | 15.47 ± 0.13^g^ |
| 130/30 | 12.91 ± 0.37^f^ |
| 160/30 | 8.10 ± 0.15^c^ |
| 100/60 | 5.96 ± 0.13^b^ |
| 130/60 | 6.22 ± 0.08^b^ |
| 160/60 | 9.07 ± 0.06^d^ |
| 100/90 | 6.16 ± 0.02^b^ |
| 130/90 | 8.77 ± 0.12^d^ |
| 160/90 | 5.18 ± 0.03^a^ |

The results obtained were expressed as mean ± SD with n=3 according to One-Way ANOVA. Different letters (a-g) designate statistically significant differences between different ozone doses and times at P < 0.05. Total phenolic content expressed as mg of catechin equivalent (CE)/g of extract.
