# Supplementary material for: The Impact of Ozone Treatment in Dynamic Bed Parameters on Changes in Biologically Active Substances of Juniper Berries
Source: PLoS One. 2015 Dec 14;10(12):e0144855. doi: 10.1371/journal.pone.0144855 (PMC4678966; doi:10.1371/journal.pone.0144855)
Supplement: S5 Table — (DOCX) [file pone.0144855.s006.docx]

**S5 Table. Antioxidant activity (DPPH, FRAP, β-carotene inhibition) of methanolic extracts and essential oils from juniper (*J. communis* (L.)) berries after ozone treatments.**

|  |  | DPPH  (mg TE/g of sample) | IC_50/DPPH_ (µg/L) | FRAP  (mM FeSO_4_ × 7 H_2_O) | β-carotene inhibition (%) |
| --- | --- | --- | --- | --- | --- |
| methanolic extract | control | 4.92 ± 0.03^a^ | 7.63 ± 0.02^b^ | 8.84 ± 0.43^d, e^ | 24.36 ± 1.07^a^ |
|  | 100/30 | 4.96 ± 0.01^a^ | 7.74 ± 0.01^d^ | 10.70 ± 0.53^g^ | 27.04 ± 1.95^a^ |
|  | 130/30 | 4.89 ± 0.02^a^ | 6.86 ± 0.00^a^ | 8.82 ± 0.22^e^ | 27.02 ± 1.31^a^ |
|  | 160/30 | 5.02 ± 0.01^a^ | 8.11 ± 0.05^f^ | 9.39 ± 0.10^f^ | 27.84 ± 1.11^a, b^ |
|  | 100/60 | 4.96 ± 0.04^a^ | 13.68 ±0.02^i^ | 5.87 ± 0.11^a^ | 31.48 ± 0.06^e^ |
|  | 130/60 | 4.79 ± 0.28^a^ | 9.39± 0.08^h^ | 7.42 ± 0.05^c^ | 29.11 ± 0.13^b, c^ |
|  | 160/60 | 4.76 ± 0.21^a^ | 7.79 ± 0.03^d^ | 8.89 ± 0.02^e^ | 26.33 ± 1.02^a, b^ |
|  | 100/90 | 5.02 ± 0.00^a^ | 7.70 ± 0.01^c^ | 8.83 ± 0.06^e^ | 30.63 ± 0.15^d^ |
|  | 130/90 | 5.00 ± 0.04^a^ | 8.68 ± 0.09^g^ | 8.39 ± 0.05^d^ | 27.68 ± 1.09^a, b^ |
|  | 160/90 | 5.01 ± 0.01^a^ | 7.85 ± 0.03^e^ | 6.90 ± 0.04^b^ | 27.48 ± 1.10^a, b^ |
| essential oil | control | 0.46 ± 0.01^c^ | 3.14 ± 0.03^f^ | 0.99 ± 0.00^e^ | 2.39 ± 0.03^d^ |
|  | 100/30 | 0.36 ± 0.02^a^ | 3.85 ± 0.04^g^ | 0.91 ± 0.02^d^ | 2.28 ± 0.06^c^ |
|  | 130/30 | 0.34 ± 0.00^a^ | 4.25 ± 0.02^h^ | 0.91 ± 0.11^d^ | 2.25 ± 0.05^c^ |
|  | 160/30 | 0.38 ± 0.00^b^ | 2.20 ± 0.03^c, d^ | 1.11 ± 0.02^e^ | 2.21 ± 0.12^c^ |
|  | 100/60 | 0.37 ± 0.01^a^ | 2.19 ± 0.08^c^ | 0.47 ± 0.02^a^ | 1.19 ± 0.08^a^ |
|  | 130/60 | 0.54 ± 0.00^d^ | 1.47 ± 0.02^b^ | 0.52 ± 0.02^b^ | 1.28 ± 0.07^a^ |
|  | 160/60 | 0.35 ± 0.00^a^ | 2.13 ± 0.00^c^ | 0.56 ± 0.01^c^ | 1.23 ± 0.05^a^ |
|  | 100/90 | 0.36 ± 0.01^a^ | 2.32 ± 0.01^e^ | 0.67 ± 0.11^c^ | 1.96 ± 0.08^b^ |
|  | 130/90 | 0.66 ± 0.01^f^ | 2.32 ± 0.05^e^ | 0.82 ± 0.15^c, d^ | 2.30 ± 0.09^c^ |
|  | 160/90 | 0.61 ± 0.01^e^ | 1.27 ± 0.08^a^ | 0.87 ± 0.16^c, d^ | 2.34 ± 0.13^c, d^ |

The results obtained were expressed as mean ± SD with n=3 according to One-Way ANOVA. Different letters (a-i) in columns designate statistically significant differences between different ozone doses and times at P < 0.05.
