# Supplementary material for: The Impact of Ozone Treatment in Dynamic Bed Parameters on Changes in Biologically Active Substances of Juniper Berries
Source: PLoS One. 2015 Dec 14;10(12):e0144855. doi: 10.1371/journal.pone.0144855 (PMC4678966; doi:10.1371/journal.pone.0144855)
Supplement: S6 Table — (DOCX) [file pone.0144855.s007.docx]

**S6 Table. LC-MS analysis of phenolics identified in methanolic extracts from juniper (*J. communis* (L.)) berries after ozone treatments.**

| Control | | | | | | After ozone treatments | | | | | |
| --- | --- | --- | --- | --- | --- | --- | --- | --- | --- | --- | --- |
| Peak | RT^a^ (min) | Λ_max_ (nm) | [M–H]^-^ | MS-MS [M–H]^-^ | Proposed molecule | RT^b^ (min) | λ_max_ (nm) | [M–H]^-^ | MS-MS [M–H]^-^ | Proposed molecule | Parameters of ozone treatment |
| 1 | 3.35 | 235, 284 | 191 | 111, 173 | Quinic acid | 3.36 | 235, 276 | 191 | 111, 173 | Quinic acid | A, B, C, D, E, F, G, H, I |
| 2 | 4.30 | 237, 276 | 205 | 111, 125, 173, 187 | Gallic acid derivative | 4.26 | 238, 276 | 205 | 111, 125, 173 | Gallic acid derivative | A, B, D, E, F, G, H, I |
| 3 | 5.68 | 237 | 405 | 359 | Unknown compound | 5.67 | 239 | 439 | 265 | Unknown compound | B |
| 4 | 5.91 | nd | | | | 5.91 | 235 | 159 | 114, 115, 129, 141 | Umbelliferone | C |
| 5 | 6.92 | 254 | 375 | 139, 201, 345, 357 | Unknown compound | 6.80 | 239 | 375 | 139, 201, 217, 357 | Unknown compound | A, B |
| 6 | 7.36 | 260, 294 | 153 | 109 | Protocatechuic acid | 7.49 | 235, 259, 294 | 153 | 109 | Protocatechuic acid | A, B, C, D, E, F, G, H, I |
| 7 | 8.96 | 238 | 447 | 401 | Unknown compound | 8.96 | nd | | | | |
| 8 | 9.31 | 238, 270 | 443 | 161, 219, 237, 281, 425 | Unknown compound | 9.43 | 235, 276 | 443 | 161, 219, 237, 425 | Unknown compound | B, C, D, E, F, G, H, I |
| 9 | 10.18 | 240, 253 | 395 | 349 | Stilbene derivative | 10.18 | 245 | 395 | 349 | Stilbene derivative | A, B, D, E, F, G, H, I |
| 10 | 10.33 | nd | | | | 10.33 | 235 | 159 | 115, 116, 132, 141 | Umbelliferone | C |
| 11 | 10.70 | 238, 280 | 289 | 205, 245 | Catechin | 10.72 | 239, 280 | 289 | 205, 245 | Catechin | B, E, F |
| 12 | 10.83 | nd | | | | 10.83 | 235, 280, 309 | 403 | 179, 357 | Caffeic acid derivative | C, E, F, G, H, I |
| 13 | 11.73 | 238, 273, 351 | 641 | 479 | Gallic acid derivative | 11.74 | 238, 271, 352 | 641 | 317, 479 | Gallic acid derivative | D, F |
| 14 | 11.80 | nd | | | | 11.80 | 235, 270 | 431 | 385 | Unknown compound | A, B, C, D, E, F, G, H, I |
| 15 | 12.98 | 238, 275, 344, 396 | 345 | 161, 301 | Coumaric acid derivative | 12.98 | nd | | | | |
| 16 | 13.03 | nd | | | | 13.03 | 235, 274 | 345 | 161, 301 | Caffeic acid derivative | A, B, C, D, E, F, G, H, I |
| 17 | 14.71 | 238, 269, 354 | 611 | 317, 449, 479 | Myricetin derivatives | 14.83 | 235, 267, 330 | 611 | 317, 449, 479 | Myricetin derivatives | A, B, C, D, E, F, G, H, I |
| 18 | 16.57 | 237, 269, 352, 396 | 641 | 317, 479 | Myricetin derivatives | 16.68 | 235 | 641 | 317, 479 | Myricetin derivatives | A, B, C, D, E, F, G, H, I |
| 19 | 18.36 | 237, 271, 348 | 655 | 331, 493 | Unknown compound | 18.38 | 235, 268, 301 | 655 | 331, 493 | Unknown compound | A, B, C, D, E, F, G, H, I |
| 20 | 20.93 | 275, 343 | 463 | 301 | Quercetin 3-O-galactoside | 21.11 | 236, 276, 344, 397 | 463 | 301 | Quercetin 3-O-galactoside | A, B, C, D, E, F, G, H, I |
| 21 | 22.75 | nd | | | | 22.75 | 235, 268, 342 | 209 | 79, 153 | 3,4-Dimethoxycinnamic acid | F |
| 22 | 25.84 | 237, 276, 341, 396 | 433 | 301 | Quercetin 3-O-arabinofuranoside | 25.93 | 235, 276, 330, 342 | 433 | 301 | Quercetin 3-O-arabinofuranoside | A, B, C, D, E, F, G, H, I |
| 23 | 27.29 | 238, 277, 305, 327 | 447 | 285, 301 | Luteolin-O-galactoside | 27.38 | 235, 277, 306, 329 | 447 | 285 | Luteolin-O-galactoside | A, B, C, D, E, F, G, H, I |
| 24 | 28.32 | 271, 297, 341, 362 | 433 | 301 | Quercetin 3- O-arabinopyranoside | 28.47 | 237, 276, 344, 396 | 433 | 301 | Quercetin 3-O-arabinopyranoside | A, B, C, D, E, F, G, H, I |
| 25 | 28.95 | nd | | | | 28.95 | 235, 279, 341, 396 | 301 | 165, 201, 229, 255, 257 | Quercetin | A, C, D, E, F, G, H, I |
| 26 | 30.33 | nd | | | | 30.33 | 235, 274 | 461 | 283, 446 | Quercetin hexose | A, C, D, E, F, G, H, I |
| 27 | 33.71 | 242, 275, 304, 328 | 417 | 285 | Kaempferol pentoside | 33.78 | 237, 276, 306, 328 | 417 | 285 | Kaempferol pentoside | A, B, C, D, E, F, G, H, I |
| 28 | 34.44 | 239, 279, 303, 325, 396 | 285 | 213, 341, 257 | Kaempferol | 34.58 | 235, 280, 304, 328 | 285 | 192, 213, 241, 267 | Kaempferol | A, B, C, D, E, F, G, H, I |
| 29 | 38.68 | 237, 270, 345, 397 | 285 | 175, 199, 217, 241, 243, 267 | Luteolin | 38.96 | 235, 310, 345 | 285 | 175, 199, 217, 241, 243 | Luteolin | A, B, C, D, E, F, G, H, I |
| 30 | 44.57 | nd | | | | 44.57 | 235 | 299 | 284 | Chrysoeriol | A, B, C, D, E, F, G, H, I |
| 31 | 45.23 | 237, 269, 337, 397 | 269 | 149, 181, 201, 225, 227, 251 | Apigenin | 45.37 | 236, 268 | 269 | 149, 201, 225 | Apigenin | A, B, C, D, E, F, G, H, I |
| 32 | 50.52 | 236, 273, 329, 397 | 537 | 375, 443 | Amentoflavone | 50.45 | 236, 274, 329 | 537 | 375, 443 | Amentoflavone | A, B, C, D, E, G, H, I |
| 33 | 55.68 | 236, 269, 334, 397 | 537 | 375, 417, 443 | Cupressoflavone | 55.57 | 236 | 537 | 375, 417, 443 | Cupressoflavone | A, B, C, D, E, F, G, H, I |
| 34 | 55.38 | nd | | | | 58.38 | 236 | 130 | 71, 85, 87, 102, 113 | Hydroxybenzoic acid | C, D, E, F, G |
| 35 | 58.53 | 236 | 225 | 141, 156, 181, 207 | Sinapic acid | 61.23 | 239, 292 | 225 | 141, 156, 181, 197 | Sinapic acid | A |
| 36 | 61.27 | nd | | | | 61.27 | 236, 293 | 130 | 59, 102, 103, 113 | Hydroxybenzoic acid | C, D, E, F, G |
| 37 | 61.37 | nd | | | | 61.37 | 237, 291 | 337 | 257, 291, 319 | p-Coumaroylquinic acid | B |

^a, b^ RT (Retention time) is an average of all RTs in analysed samples.

A – 100/30; B – 130/30; C – 160/30; D – 100/60; E – 130/60; F – 160/60; G – 100/90; H – 130/90; I – 160/90

nd – not detected
